# Bentall-De Bono Procedure as a Treatment for Stanford Type A Chronic Aortic Dissection

**DOI:** 10.7759/cureus.92823

**Published:** 2025-09-21

**Authors:** Jorge Armando Ramírez-López, Mario Gómez-Sánchez, Natalia Dettmer-Andrade

**Affiliations:** 1 Surgery, Hospital Central Sur de Alta Especialidad (Central South High Specialty Hospital), Mexico City, MEX; 2 Cardiothoracic Surgery, Instituto Nacional de Ciencias Médicas y Nutrición Salvador Zubirán (National Institute of Medical Sciences and Nutrition Salvador Zubirán), Mexico City, MEX; 3 Surgery, Facultad Mexicana de Medicina Universidad La Salle (Mexican School of Medicine, La Salle University), Mexico City, MEX

**Keywords:** aortic root dilation, bental-de bono, chronic aortic dissection, - marfan syndrome, stanford type a dissection

## Abstract

Marfan syndrome is a connective tissue disorder with multisystemic involvement, in which cardiovascular complications, particularly aortic dissection, are the leading cause of mortality. We report a case of a 35-year-old woman with known Marfan syndrome who complained of chest pain in the last 4 months and was found to have a chronic Stanford type A aortic dissection and severe aortic insufficiency. Surgical repair with a Bentall-De Bono procedure was successfully performed. This case highlights the importance of early recognition of chronic dissection in Marfan patients and the effectiveness of surgical management using the current gold standard approach.

## Introduction

Marfan syndrome is an autosomal dominant connective tissue disorder caused by mutations in the *FBN1 *gene, which encodes fibrillin-1, a key component of extracellular microfibrils. Patients present with bone, muscular, and cardiovascular abnormalities. Approximately 90% of individuals with Marfan syndrome develop cardiovascular manifestations, and aortic complications such as thoracic aortic dissection and progressive dilation of the aortic root are suggested to be the primary causes of morbidity and mortality [1]. Among these, dissection of the ascending aorta represents a surgical emergency due to the high risk of rupture and mortality. Surgical replacement of the dilated aortic root and ascending aorta demonstrates a significant increase in life expectancy in patients with Marfan syndrome [2].

This report presents a case of chronic Stanford type A aortic dissection in a hemodynamically stable Marfan patient, who presented at the National Institute of Medical Sciences and Nutrition Salvador Zubirán (INCMNSZ), a major reference center in Mexico City, where, through a directed physical examination and support of imaging studies, her admission for surgical resolution was determined. The report highlights the importance of early recognition and surgical intervention to improve outcomes.

## Case presentation

We present the case of a 35-year-old female patient with a family history of a mother with Marfan syndrome who underwent aortic repair due to aortic dissection. She had a personal history of type 2 diabetes, well-controlled hypertension, and Marfan syndrome diagnosed by genetic testing. She attended an internal medicine consultation on April 30, 2024, reporting sporadic stabbing/pressing chest pain since January 2024, of variable intensity, not associated with physical activity. The pain occurred at rest, was of variable duration - from 1 to 20 minutes - and sometimes accompanied by dyspnea on minimal exertion. Physical examination revealed a reinforced second sound and a grade 4/6 diastolic murmur in the aortic area radiating to the apex. An Austin Flint murmur was absent.

A transthoracic echocardiogram was performed for further assessment on May 24, 2024. It revealed a dissection flap in the aortic root, ascending aorta, and descending aorta; dilation of the ascending aorta to 60 mm in its largest diameter; and severe aortic insufficiency with eccentric regurgitant flow, regurgitant volume of 44 mL, effective orifice area (EOA) of 0.31 cm², and a vena contracta of 0.7 cm. She was therefore referred to the emergency service of the INCMNSZ, where, at the time of assessment, she had stable vital signs: heart rate 90 bpm, respiratory rate 17 rpm, right arm blood pressure 121/51 mmHg, and left arm blood pressure 117/51 mmHg. She was without hemodynamic instability and denied chest pain, intermittent claudication, headache, cyanosis, fatigue, or palpitations. On physical examination, there was no Musset sign, but Corrigan’s pulse was present.

**Figure 1:**
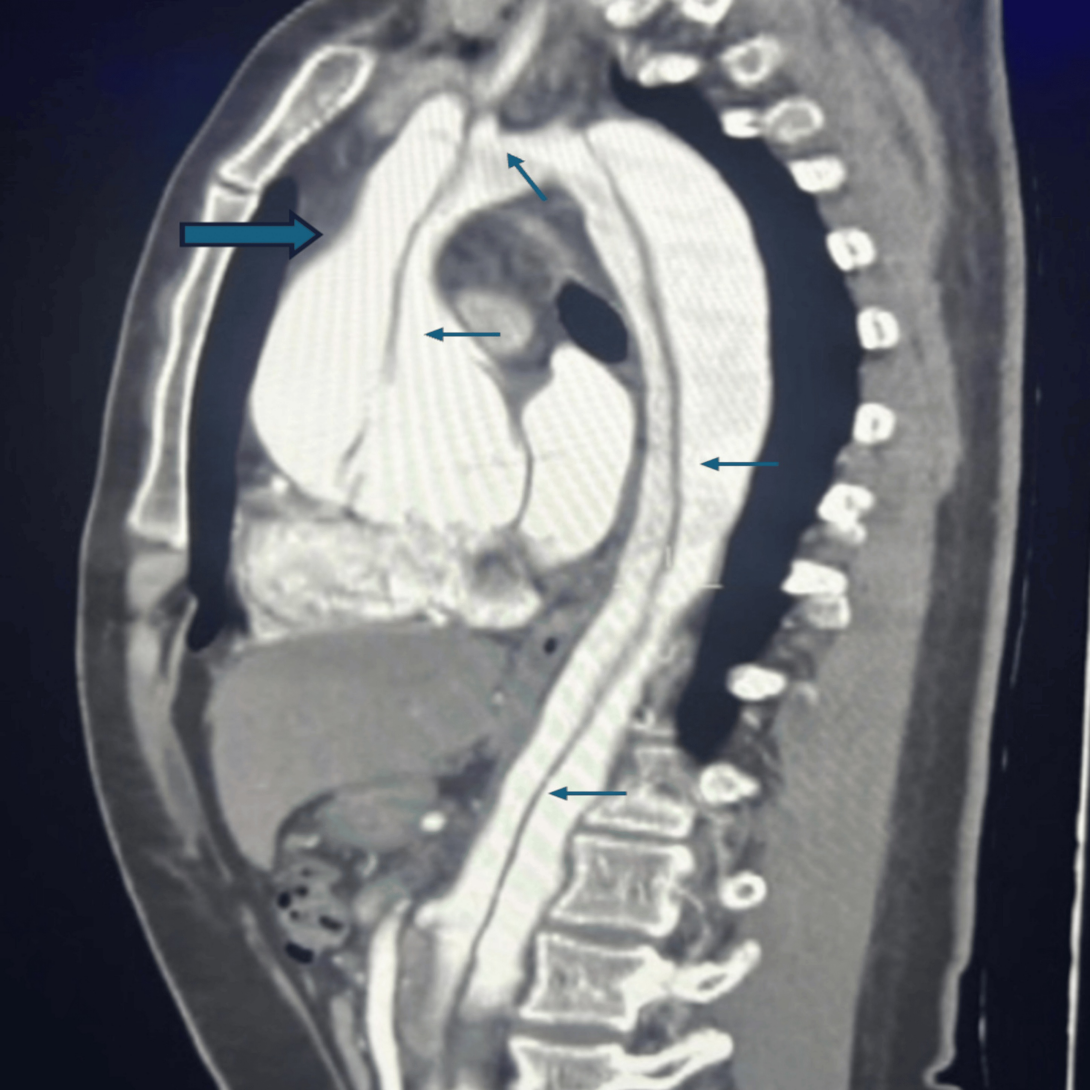
Thoracoabdominal angiotomography showing dilation of the aortic root (thick arrow) and aortic dissection involving the root, trunk, and arch, and descending aorta (thin arrows)

Angiotomography of the chest and abdomen was performed (Figure 1), revealing aortic dissection involving the aortic root and supra-aortic branches supplied by the false lumen, extending to the iliac bifurcation, with the false lumen also supplying the supra-aortic vessels and the left renal artery. A diagnosis of chronic Stanford type A aortic dissection with severe aortic insufficiency was established, and the cardiovascular surgery team was consulted. The team admitted the patient for inpatient management, including monitoring, blood pressure and heart rate control, and analgesia, with plans for subsequent surgical intervention.

**Figure 2:**
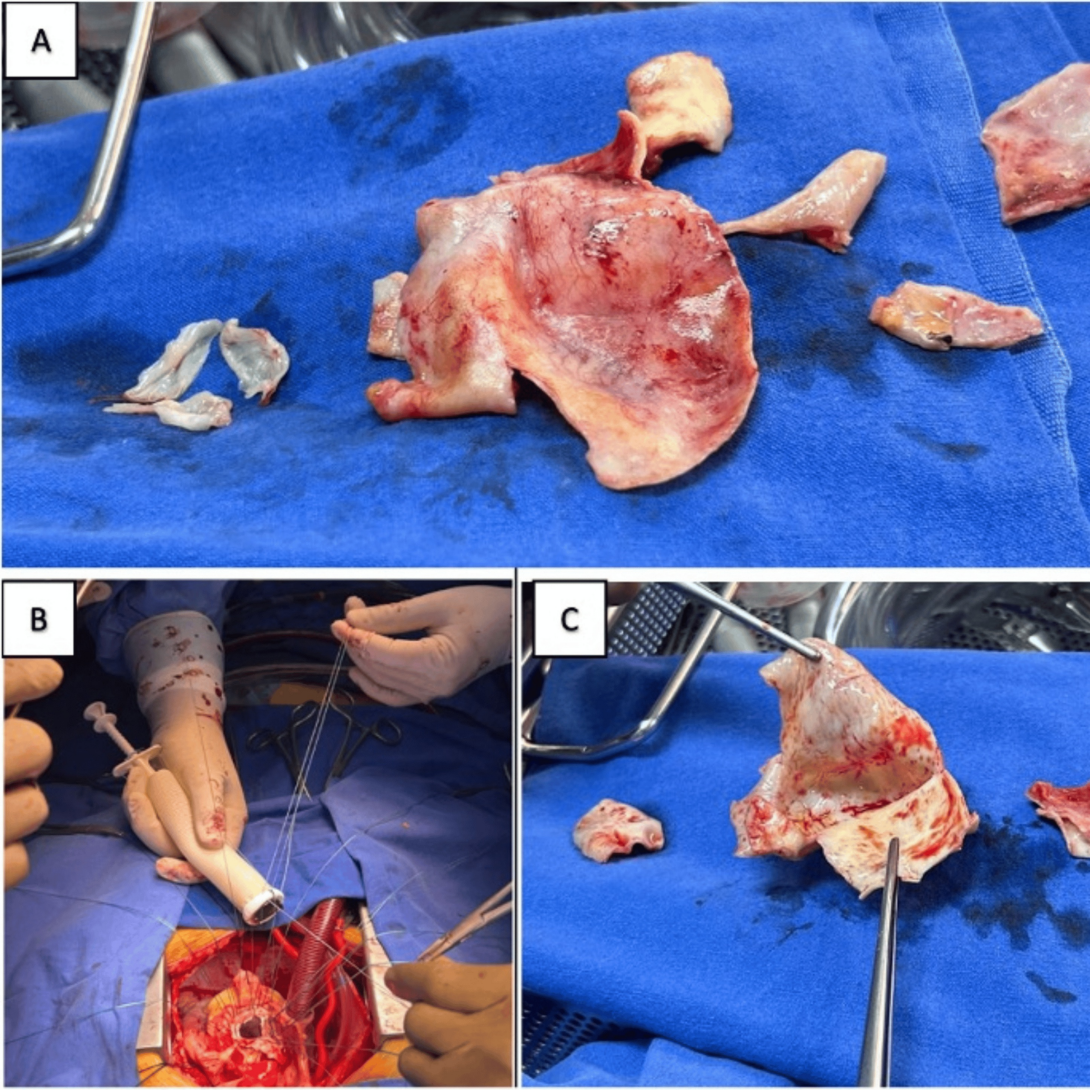
Relevant surgical images A: Aortic valve leaflets and aortic root resected, in which the increase in the diameter is clearly evident, corresponding to aortic dilation. B: Dacron graft (Medtronic, Inc., Minneapolis, USA) with St. Jude mechanical 23 aortic prosthesis (Abbott Laboratories, Abbott Park, USA) at the moment of colocation. C: Rupture site and dissection path.

Based on the availability of operating rooms, surgery was scheduled for May 30, 2024. The technique used was replacement of the ascending aorta with a Dacron graft (Medtronic, Inc., Minneapolis, USA), aortic valve replacement with a 23 mm St. Jude mechanical prosthesis (Abbott Laboratories, Abbott Park, USA), and coronary artery reimplantation (Bentall-De Bono technique) (Figure 2), with an aortic cross-clamping time of 115 minutes and 160 minutes of extracorporeal circulation. The intraoperative transesophageal echocardiogram showed a normally functioning mechanical aortic prosthesis and a thoracic aortic graft with adequate positioning and without dysfunction.

Following surgery, the patient was transferred to the intensive care unit for monitoring with vasopressor support. Upon admission, she was on aminergic support based on norepinephrine at 0.14 mcg/kg/min and dobutamine at 3 mcg/kg/min, with a pacemaker set to demand mode. Invasive hemodynamic monitoring was performed using a Swan-Ganz catheter, requiring administration of albumin and crystalloid solution, which allowed for a gradual reduction of vasopressor doses until their discontinuation at 1:00 a.m. the following day. Similarly, once the sedative effects had worn off on the same day as the surgery, invasive mechanical ventilation was successfully discontinued, and the patient was maintained on nasal cannula oxygen support.

**Figure 3:**
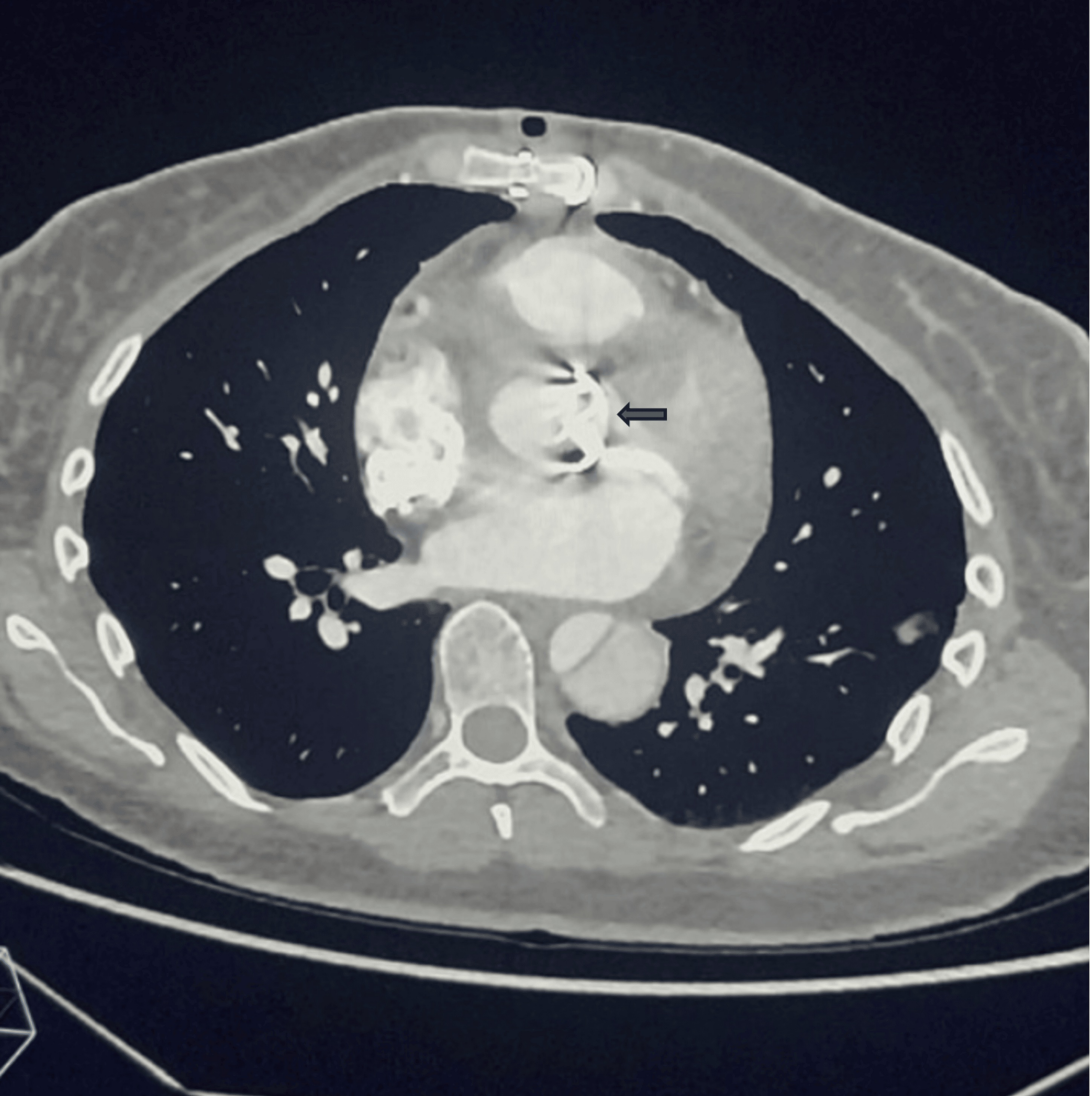
Follow-up CT scan. A properly positioned mechanical aortic prosthesis is observed (arrow).

During the patient's stay in the ICU, where she stayed for 3 days, laboratory results remained stable, with a maximum hemoglobin decrease of 3 grams (10.7 g/dL) compared to the baseline level (14.5 g/dL). She was maintained on a systolic blood pressure of 100 mmHg and a heart rate between 60 and 80 bpm for graft protection. A follow-up non-contrast chest CT scan was performed (May 30, 2024), reporting post-surgical changes due to the replacement of the ascending aorta with a Dacron graft, aortic valve replacement with a mechanical prosthesis, and reimplantation of coronary arteries. Hematomas were observed adjacent to the anterior edge of the graft and the right atrium, without evidence of active bleeding in that area (Figure 3). The rest of the aortic dissection showed no changes in extent or behavior compared to the previous study, and there was no vascular compromise of the limbs or organs.

Based on adequate clinical progress, the patient was transferred to the general ward on June 3, 2024, where she remained hospitalized for 10 days under postoperative monitoring. Oral anticoagulation therapy was initiated, and upon achieving target international normalized ratio (INR) levels, discharge home was decided on June 13, 2024, due to clinical improvement. Treatment of the remaining descending aorta will be planned during a second hospitalization.

## Discussion

Aortic dissection is characterized by the separation of the media layer of the aortic wall, most often due to a tear in the intimal layer following a weakening process [3], as seen in this case with Marfan syndrome. The Stanford classification is commonly used to categorize aortic dissections into type A and type B, based on the segments of the aorta involved. Type A involves dissection of the ascending aorta, with or without involvement of the descending aorta, while type B is limited to the descending aorta. Type A is more frequently observed, accounting for up to 63.3% of cases [4].

Aortic dissection can be classified according to the timing of symptom onset into distinct phases: hyperacute (first 24 hours), acute (up to 14 days), subacute (15 to 90 days), and chronic (more than 90 days after symptom onset) [5]. The majority of cases present during the acute phase and are associated with high mortality rates. It is estimated that fewer than 10% of cases progress to the chronic phase [6]. However, due to advances in early and effective diagnostic strategies, improved risk stratification, and timely surgical interventions when indicated, survival rates for patients experiencing acute aortic dissection have improved significantly in recent years [7].

Resolution of the acute event leads the patient to the management of the chronic phases, during which degeneration of the false lumen and aneurysm formation commonly occur, often necessitating surgical, endovascular, or hybrid interventions for management [8]. This is exemplified in the present case, where the patient reports episodes of progressive chest pain, aborted with self-medication, for up to four months, consistent with the chronic phase of dissection. The prevalence of chronic aortic dissection remains uncertain and difficult to estimate; however, it contributes to increased healthcare costs due to ongoing surveillance and the need for interventions.

Assuming a conservative incidence rate of four to six cases per 100,000 individuals per year, and an average survival of seven years, the prevalence is estimated to be between 28 and 42 cases per 100,000, corresponding to approximately 92,000 to 138,000 survivors of acute dissection in the United States [8]. This underscores the clinical importance of understanding long-term management and individualized surgical decision-making due to the low prevalence of cases like this.

There are both surgical and non-surgical treatments available for vascular complications associated with Marfan syndrome. Surgery is recommended when the aortic diameter is ≥5 cm, when aortic dilation progresses to ≥0.5 cm per year, or when there is a family history of aortic repair [1], as illustrated in the present case, where the patient’s mother underwent aortic surgery due to Marfan syndrome with aortic dissection. Although there are studies comparing conservative aortic valve-sparing procedures, such as the David procedure, with aortic valve replacement using a prosthetic valve, both approaches are considered appropriate surgical options for aortic root replacement in the context of acute Stanford type A aortic dissection [9].

While valve-sparing techniques such as the David procedure are viable options for aortic root replacement in patients with connective tissue disorders, the Bentall-De Bono procedure was preferred in this case. This choice was influenced principally by the chronic nature of the dissection, which increased the risk of future valve dysfunction, thus prioritizing long-term durability. Additionally, the need for reintervention after valve-sparing procedures is reportedly higher in Marfan patients compared to the general population, reinforcing the decision for a composite graft with prosthetic valve replacement. The Bentall-De Bono procedure remains the current gold standard for aortic dissections involving the aortic root and valve [10]. This is why this surgical procedure was chosen for our patient.

The patient’s prognosis following the Bentall-De Bono procedure is generally favorable when performed electively in stable chronic dissection cases, particularly in specialized centers. However, the chronic nature of the dissection and the underlying Marfan syndrome necessitate ongoing vigilance. Potential complications include prosthetic valve thrombosis, pseudoaneurysm formation at anastomotic sites, and continued false lumen perfusion distal to the repair site. Therefore, long-term outcomes depend not only on the technical success of surgery but also on sustained multidisciplinary follow-up [11].

## Conclusions

Aortic dissection, particularly involving the ascending aorta, remains a life-threatening condition requiring timely diagnosis and intervention. While acute presentations are more common, this case highlights the importance of considering chronic Type A dissection in patients with known connective tissue disorders who present with non-specific or subacute symptoms. The successful use of the Bentall-De Bono technique reinforces its role as the standard surgical treatment in cases with aortic root and valve involvement. Long-term surveillance and individualized planning remain essential in patients with residual dissection and systemic connective tissue disease such as Marfan syndrome.
